# Percyanide of Mercury in Syphilitic Ulceration of the Tongue

**Published:** 1855-10

**Authors:** 


*Percyanide of Mercury in Syphilitic Ulceration of the Tongue.*—Mr. Wormald, at St. Bartholomew's Hospital, has recently been employing a saturated solution of the bicyanide of mercury, as an application to syphilitic ulcerations, abrasions, etc., on the tongue. Without speaking very enthusiastically respecting it, he states that he has obtained more satisfactory results from it than from any remedy he had previously employed. The solution is painted over the affected part, care of course being taken that the patient do not swallow any quantity of it. The extremely intractable nature of this form of syphilis is matter of general remark.—*Medical Times & Gazette, July,* 1855.

